# Heavy Metals in Four Marine Fish and Shrimp Species from a Subtropical Coastal Area: Accumulation and Consumer Health Risk Assessment

**DOI:** 10.3390/biology11121780

**Published:** 2022-12-07

**Authors:** Mohammad Belal Hossain, Nurer Zaman Bhuiyan, Abul Kasem, Md. Kamal Hossain, Salma Sultana, As-Ad Ujjaman Nur, Jimmy Yu, Mohammed Fahad Albeshr, Takaomi Arai

**Affiliations:** 1 Department of Fisheries and Marine Science, Noakhali Science and Technology University, Noakhali 3814, Bangladesh; 2 School of Engineering and Built Environment, Griffith University, Brisbane, QLD 4111, Australia; 3 Independent Researcher, Mustankivenkatu 2C 38, 00980 Helsinki, Finland; 4 Soil and Environment Research Section, BCSIR Laboratories Dhaka, Bangladesh Council of Scientific and Industrial Research (BCSIR), Dhaka 1205, Bangladesh; 5 Department of Zoology, College of Science, King Saud University, P.O. Box 2455, Riyadh 11451, Saudi Arabia; 6 Environmental and Life Sciences Programme, Faculty of Science, Universiti Brunei Darussalam, Jalan Tungku Link, Gadong BE1410, Brunei

**Keywords:** toxic metals, accumulation, fish, health risk, consumers

## Abstract

**Simple Summary:**

This study assessed the toxic metal levels in four species of fish and shrimp (*Labeo bata*, *Sillaginopsis panigus*, *Platycepalus fuscus*, and *Penaeus monodon*) and the associated health risks to consumers. The findings revealed that Pb, Cu, and Zn exceeded the national recommended guidelines, indicating possible risks to human health. Shrimp species had higher levels of metals than fish species. However, the results of some risk assessment indices demonstrated no possible carcinogenic risks to consumers.

**Abstract:**

Trace-element or heavy-metal pollution has emerged as a serious concern in terms of both environmental and human health issues. This study measured six trace and toxic heavy metals (Pb, Cd, Cr, Ni, Cu, and Zn) in four marine fish and shrimp species to assess their accumulation levels and evaluate the risks to human health. The mean concentrations of the metals in fish and shrimp species (*Labeo bata*, *Sillaginopsis panijus*, *Platycepalus fuscus*, and *Penaeus monodon*) followed the decreasing order of Zn (40.8 ± 9.7 μg/g) > Cu (17.8 ± 7.1 μg/g) > Pb (6.2 ± 1.8 μg/g) > Ni (0.4 ± 0.3 μg/g) > Cd (0.06 ± 0.02 μg/g > Cr (below detection level). Among the metals, only Pb in finfish and Pb, Cu, and Zn in shrimp samples exceeded the national recommended limits, representing possible risks to consumers. The mean metal concentrations in the studied fish/shrimp species followed the descending order of *P. monodon* > *S. panijus* > *P. fuscus* > *L. bata*, which implies that bottom dwellers and omnivores had higher levels of metals. However, the estimated daily intake (EDI) concentrations of Zn and Cu for the studied species were lower than the RDA (Recommended Daily Allowance). In addition, the Target Hazard Quotient (THQ) and hazard index (HI or TTHQ) values for all species were < 1, indicating that consumers might not experience carcinogenic health risks. A strong significant (*p* < 0.05) correlation between Cu and Pb (*r* = 0.623) and Zn and Cu (*r* = −0.871) indicated they were from the same source of origin. Cluster analysis (CA) and principal component analysis (PCA) demonstrated possible anthropogenic sources of toxic metals in the study area, specifically industrial wastes and agricultural chemicals.

## 1. Introduction

Toxic metals, mainly heavy metals, pose a serious threat worldwide due to their accumulative properties, inherent persistence, non-biodegradability, and harmful effects. [1]. Fish and shellfish are considered to be the most frequent bioindicators for monitoring contaminants such as heavy metals (e.g., Cd, Cr, and Pb) due to their higher position in the aquatic food chain. Additionally, people consume them as a major source of protein [2]. Metals are distributed throughout the water column, deposited in sediments, and consumed by fish in the aquatic environment. If large amounts of metals are taken in or if they accumulate above a certain threshold, they cause random interactions with cellular biomolecules such as chemicals and proteins, resulting in substances that can damage the metabolism of cells [3].

Although metals are naturally occurring elements in the environment, human activities, such as rapid industrialization and urbanization, significant land-use changes, and increased soil runoff, can increase their concentrations [4]. In Bangladesh, the majority of tannery, textile, electroplating, mining, dyeing, printing, photography, and pharmaceutical industry effluents are dumped directly into rivers. River water flow that contains toxins contaminates coastal ecosystems [5]. As a result, the levels of hazardous metals in these water sources surpass WHO and national standard recommendations [6]. The uptake, retention, and bioaccumulation of metal pollutants in fish tissues may be influenced by a variety of parameters, including sex, age, season, breeding period, the diversity of food habitats, pollutant exposure, and evolutionary changes in regulatory mechanisms [7].

Contamination by toxic metals not only endangers aquatic life but also poses a serious risk to human health. For example, excess Cr, Pb, and Cd contamination can be toxic to living organisms, and long-term exposure can cause injury or death [8]. Even at modest doses of about 1 mg/kg, long-term Cd consumption has been linked to illnesses such as prostatic growth, cellular collapse in the lungs, bone fractures, and kidney dysfunction [4]. Humans can be affected by severe Pb exposure, which can result in disorders of the nervous system, skeletal hematopoiesis, and even mortality [9]. It has been established that an excessive amount of Cr is carcinogenic to humans [10]. Ingesting a high amount of copper, which is typically found in drinking water, can harm the kidneys and liver and induce vomiting, nausea, abdominal pain, and diarrhea [11]. Zn and Cu are found commonly in the environment and spread there by means of natural occurrences. Both metals are necessary for the body’s regular functions, including those of the thyroid and immune system, blood coagulation, sensations of taste and smell, and wound healing [6,7]. Although humans can tolerate relatively high concentrations of these metals, excessive ingestion of Cu and Zn can nevertheless have serious negative effects on health. Long-term exposure to Cu and Zn can result in eye irritation, as well as headaches, stomachaches, vomiting, and diarrhea. For instance, taking too much zinc (>40 mg/day) might cause headaches, nausea, diarrhea, and abdominal cramps [10].

The coastal areas of the subtropics, including Bangladesh, are rich in fishery resources. However, recent rapid urbanization, industrialization, and the growth of aquaculture activities in this region are rapidly depleting these healthy ecosystems. The central coast of Bangladesh, including the Lakshmipur, Noakhali, and Feni districts, which contact the Bay of Bengal, is no exception. The Bay of Bengal is said to be polluted by oil and chemical waste discharged from domestic and foreign ships. Tidal waves flush the catchment area of the central coast, which has a strong influence on pollution levels [12,13]. Much research has been carried out to evaluate the levels of heavy metals in fish and the risk of human exposure [5,14,15,16]. In addition, several studies have looked into heavy-metal contamination and pollution load estimation in shrimp from the island of Saint Martin [10,17], the Khuln-Satkhira region [18], and Cox’s Bazar region [1] in Bangladesh. However, published reports concerning toxic-heavy-metal contamination and the associated health risks through the consumption of *L. bata*, *P. fuscus*, *S. panijus*, and *P. monodon* from the central coast of Bangladesh are scant. Therefore, the aim of the present study was (i) to measure the accumulation levels of toxic metals in the muscles of some commercially important marine fishes (*Labeo bata*, *Sillaginopsis panijus*, and *Platycephalus fuscus*) and shellfish (*Penaeus* monodon) from the central coastal region of Bangladesh and (ii) to evaluate the health risk to consumers.

## 2. Materials and Methods

### 2.1. Study Area

**Figure 1:**
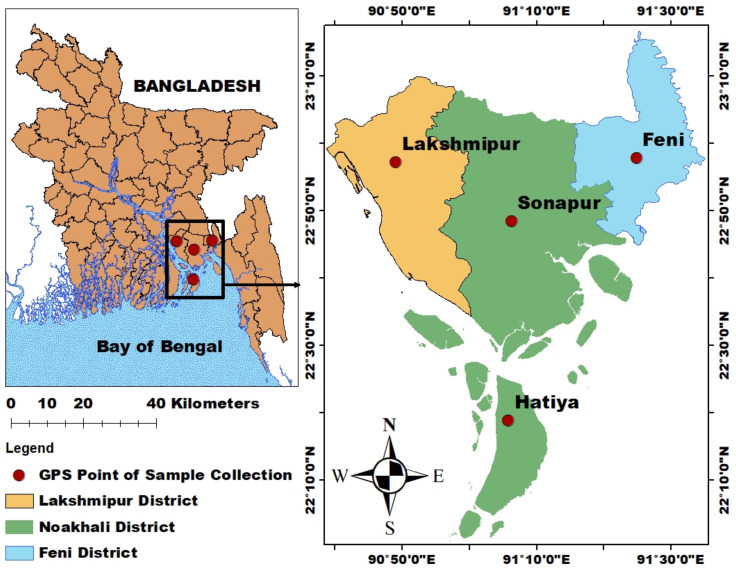
Sampling locations along the northern Bay of Bengal Coast.

The research was carried out in a subtropical coastal area in Bangladesh, encompassing the regions of Hatiya (22.282405 N, 91.09694 E), Lakhsmipur (22.944674 N, 90.828191 E), Sonapur (22.823557 N, 91.100674 E), and Feni (22.940878 N, 91.406666 E). The study area is grouped into four regions that cover almost all of the important places in Greater Noakhali (Figure 1). The present study area is located in the upper and lower tidal channels of the Meghna River estuary, which is the largest estuarine ecosystem in Bangladesh. The mean temperatures range from 12 °C (December–February) to 34 °C (April–June) with an average annual rainfall of 2980 mm. Southeast monsoon winds with cloudiness, moisture, heavy rainfall, thunderstorms, cyclones, and occasional storm surges characterize the monsoon season (June–October). The sources of pollution in these areas are mainly industrial wastes, agricultural chemicals, fecal waste, oil spills from fishing and passenger boats, and other small industrial effluents. Water quality parameters are included in Table S1.

### 2.2. Sample Collection

From March to September 2018, four species (*Labeo bata*, *Sillaginopsis panigus*, *Penaeus monodon*, and *Platycepalus fuscus*) were obtained from the Nothern Bay of Bengal by fishermen from Feni, Sonapur, Laxmipur, and Hatiya (Figure 1). In total, 48 samples of fish and shrimp samples (12 samples for each species) were collected from the area for metal analysis. The fish samples obtained were immediately kept in pre-cleaned polythene bags, sealed, labeled and kept in ice boxes for transport to the laboratory.

### 2.3. Reagents

Throughout the experiment, analytical-grade chemicals and reagents were utilized (Sigma- Aldrich, Merck, Taufkirchen, Germany). Chemicals and reagents used in this study include de-ionized water (resistivity > 18 MΩ·cm, manufactured using an E-pure system, Thermo Scientific, Waltham, MA, USA), nitric acid (67%, BDH), hydrogen peroxide (30%, Sigma Aldrich), HCl (37%, Loba, Mumbai, India), and certified reference material (CRM) for Ni (1000 ± 3 mg/kg; Cica-Reagent; CAS-285772B), for Pb, Cd, Cr, Ni, and Zn (1000 ± 3 mg/kg; Sigma Aldrich, Honeywell Specially Chemicals Seelze GmbH, Seelze, Germany), and for SRM 2976-Muscle tissue (Sigma-Aldrich Chemie GmbH, Taufkirchen, Germany). All chemicals and reagents were used without further purification, and the working standard was prepared from stock CRM following the standard procedure.

### 2.4. Sample Preparation, Digestion, and Metal Extraction

**Figure 2:**
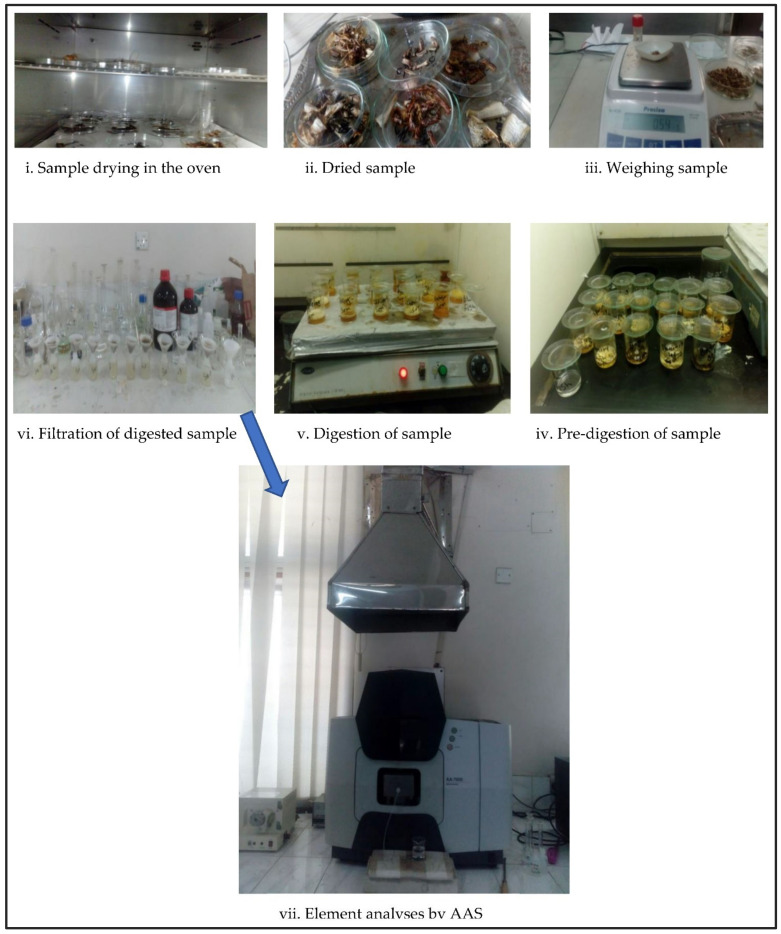
Main steps of heavy-metal analyses using AAS.

After transfer to the laboratory, all fish and shrimp samples were washed with deionized water. The muscles of collected fish and shrimp were dried at 105° in the oven to fully remove the moisture from the flesh (Figure 2). Then, the microwave digestion (AOAC Method 999.10) process was followed to prepare the samples. In this process, 0.50 g of dried fish sample was homogenized, precisely weighed, and then added to PTFE-TFM digestion containers. In the pre-digestion stage, only concentrated HNO_3_ was added and heated slowly. After the pre-digestion process, the samples were kept on the hot plate of the fume chamber and digested at 80–90° for 2–3 h. Each time, the following reagents were added: 5.0 mL of concentrated nitric acid, 2.0 mL of hydrogen peroxide, and 1.0 mL of water. The only components of the analytical reagent blanks, which were likewise created, were the acids and water without a fish sample. Once sealed, the vessels were put into Rotor 9 for microwave digestion (START D Microwave Digestion System, Milestone, Sorisole (BG), Italy). The digestive fluids were transferred to a 50.0 mL volumetric flask after the digestion process. After the digestion process, the samples were cooled under a fume hood and then filtered with Whatman Filter No. 44. Finally, the filtered samples were used for metal analyses. Six trace and toxic heavy metals (Pb, Cd, Cr, Ni, Cu, and Zn) were determined using an atomic absorption spectrometer (AA-7000, Shimadzu, Kyoto, Japan) fitted with a single-element hollow cathode lamp, and the concentration of each element was determined using AAS (AA-7000F/AAC Dual Atomizer System (Auto Atomizer Changer/and Graphite Furnace Atomizer) with electrothermal atomization, which is suitable for such analysis. The analysis of the Certified Reference Material (CRM) for these experiments was taken into account for the quantification of each metal, and their recovery rates are stated in Table 1. To determine standard deviations, a calibration graph showing the correlation coefficients between the relevant elements was created. All of the elements’ accuracies in the data were verified using certified reference materials from Sigma-Aldrich, Taufkirchen, Germany. SRM 2976-Muscle tissue, certified reference material from the National Institute of Standards, was examined to ensure the accuracy, sensitivity, and precision of the analytical technique of AAS (NIST). The limits of detection (LODs) of the AAS system for Pb, Cd, Cr, Ni, Cu, and Zn) were 0.02, 0.01, 0.02, 0.01, 0.2, and 0.1 ppm, respectively. Thus, the following formula was used to determine the concentration of the examined metals: where sample volume and weight are the primary dilution factor (PDF), and sample secondary volume and weight are the secondary dilution factor (SDF), the calculation of the concentration of trace metals = (Reading − Blank reading)*PDF*SDF.

### 2.5. Quality Control

To prevent contamination, transparent, powder-free latex gloves and lab coats were worn when handling the samples. Glassware was thoroughly cleaned using distilled water and a chromic acid solution. Blank determinations were employed to obtain accurate measurements. The data were statistically analyzed using the least-squares technique and straight-line approximation. When determining the concentrations of various elements, the blank samples were used to make any necessary changes. To ensure the accuracy and precision of data, certified reference material (SRM 2976) from Sigma-Aldrich Chemie GmbH (Taufkirchen, Germany) was used. The obtained values were compared with reference values and showed good agreement. The percentages of recovery were between 93 and 106%. The relative standard deviation (RSD) was ≤ 11%.

### 2.6. Health Risk Assessment of Fish and Shrimp

#### 2.6.1. Estimated Daily Intake Assessment

The following formula was used to determine the estimated daily intake (EDI) of the metals based on the metal concentration, daily food consumption, and consumer body weight [11,19]:

EDI = (DFC × MC)/BW(1)

where DFC is the amount of food (fish) consumed per day, and MC is the mean metal concentration in fish/shrimp muscle tissue. Based on the “Report of the household income and expenditure survey 2015”, in this study, we took into account an average of 49.5 g of daily fish ingestion for a Bangladeshi adult person (60 kg).

#### 2.6.2. Non-Carcinogenic Risk Assessment: Target Hazard Quotient

The Target Hazard Quotient (THQ) was calculated using the ratio of EDI to the oral reference dose (RfD). A value of the ratio < 1 implies a non-significant risk effect. The THQ formula is expressed as follows [5].

(2) $$\mathrm{THQ}=\frac{\mathrm{ED}\;\times \;\mathrm{Ep}\;\times \;\mathrm{EDI}}{\mathrm{RfD}\;\times \;\mathrm{AT}}\times 10^{-3}$$

where, ED = the exposure duration (65 years) (USEPA, 2008); Ep = the exposure frequency (365 days/year), AT = the average time for a non-carcinogenic element (Ed × Ep), EDI= estimated daily intake and RfD = average reference dose (mg/person/day) for each metal, e.g., for Pb and Cd, the values are 0.002, 0.001 [12,20].

Exposure to two or more metal pollutants may result in additive and/or collaborating effects. So, the cumulative health risk is evaluated by summing THQ, which is also known as the hazard index (HI), as follows:

TTHQ (HI) = THQ (Metal 1) + THQ (Metal 2) + … + THQ (Metal n)(3)

A greater value of TTHQ (HI) indicates a greater concern. A TTHQ (HI) above 1 indicates an adverse human health effect and suggests the need for possible remedial action [9].

### 2.7. Statistical Analysis

Statistical methods (descriptive statistics, e.g., mean, SD, ANOVA, and correlation) were used to assess complex ecotoxicological processes by showing connections and interdependencies between variables and their relative weights. Mathematical calculations (contamination assessment indices) were performed with the use of Microsoft Excel version 10. Multivariate and univariate statistical analyses, such as principal component analysis (PCA), cluster analysis (CA), and a correlation matrix (CM), were carried out using free statistical software, PAST (version 3.0). Additionally, the site map was tailored by Arc GIS (v. 10.3) software.

## 3. Results and Discussion

### 3.1. Heavy-Metal Concentration in Fish and Shrimp

The studied fish and shrimp specimens included a wide range of heavy-metal concentrations (Table 2). The loads of six selected heavy metals (Pb, Cd, Cr, Ni, Cu, and Zn) followed the decreasing order of Zn (40.8 ± 9.7 μg/g) > Cu (17.8 ± 7.1 μg/g) > Pb (6.2 ± 1.8 μg/g) > Ni (0.4 ± 0.3 μg/g) > Cd (0.06 ± 0.02 μg/g). The concentration of Cr was below the detection level in all of the experimented samples. Among the metals, Pb concentrations exceeded the recommended guidelines. The findings, however, revealed significant variations in the four measured metal contents among the four species at a 99% confidence interval (*p* < 0.01). It has been demonstrated through experimentation that several biotic and abiotic factors, such as the fish habitat, eating behavior, age, sex, body weight, physiological state, water temperature, pH, etc., can affect the variation in heavy metal accumulation in fish. Benthic or bottom-dwelling fish typically accumulate more metals than their surface-dwelling counterparts because they take up metals from both water and sediments [12,20,21,22]. The level of heavy-metal contamination increases with fish size and age and is higher in females than in males and higher in predators than in herbivores [22]. Once more, the accumulations are lowest in the muscle and are highest in the liver and gills. The levels also vary depending on the study area because higher metal levels have been seen in regions with growing populations, industries, traffic, and agricultural activities.

The findings of this study were compared to earlier research on the presence of heavy metals in marine and estuarine fish and shellfish from different regions of Bangladesh, as shown in Table 2. In this study, the average metal level was high in *P. monodon*, followed by *S. panijus*, *P. fuscus*, and *L. bata.* The maximum loads of Zn (49.98 µg/g), Cu (42.33 µg/g), and Pb (7.24 µg/g) were recorded in *P. monodon*, whereas Ni (0.81 µg/g) and Cd (0.08 µg/g) were higher in *L. bata* and *P. fuscus*, respectively. The highest levels of metals were recorded in omnivores (*P. monodon)* and carnivores *(P. fuscus)*, and the lowest levels were in herbivores *(L. bata).* Researchers have discovered that the feeding habits of fish have a significant impact on the amount of metal they absorb. For instance, carnivorous fish have higher metal concentrations than herbivores and omnivores. In addition, the fish habitat may potentially be a factor in the metal concentrations in fish. Hossain et al. [26] documented higher metal concentrations in benthic fish than in demersal fish. The variation in metal loads in the fish and shellfish of this study might be due to the different ecological needs, metabolism, and feeding patterns of the examined fishes [27,28,29]. The toxicity or bioavailability of metals is influenced by physical–chemical factors, chemical species, and the living things themselves. In a slightly acidic environment (pH = 5.0), metal solubilities were higher, and they sharply rose when the pH was below 4. The toxicity of metals is also said to rise with rising temperatures. The rate of oxygen consumption rose by about 34% with a temperature increase of 4 degrees Celsius (from 20 to 24 degrees C), which enhanced copper toxicity by around 7% [28].

From the comparison in Table 2, it was observed that the examined fish (*L. bata*, *P. fuscus*, and *S. panijus*) had higher concentrations of Pb than other fish in Bangladesh [12,20,21,22]. Although Sultana et al. [1] recorded higher Pb in *P. monodon* than the present study, Pb was found to be lower in other shellfish, e.g., *Macrobrachium rosenbergii*, *Metapenaeus dobsoni*, *P. sculptilis*, and *P. versicolor* [5,22]. However, Pb, in all of the examined fish and shrimp of this study, exceeded the guideline for Bangladesh (0.3 µg/g for fish and 0.5 µg/g for shellfish) [24]. Pb is responsible for many health problems, such as neurotoxicity, nephrotoxicity, etc. [1]. However, *P. monodon* is a bottom-dwelling shellfish and grazes on sediments. Therefore, sediments could be a major source of Pb in fish and shellfish [18].

The content of Cd in fish and shellfish muscle in this study was similar to the findings of Tahity et al. [12] and Sultana et al. [1]. In contrast, the Cd load in the present studied species was found to be lower than the findings of Islam et al. [21] and Baki et al. [5]. However, the load of Cd in all of the species did not cross the tolerance level or guidelines for Bangladesh (Table 1). Cd is a chronic-toxicity-producing metal that naturally exists in the ecosystem in a very low quantity. Aquatic ecosystems may have Cd from industrial waste, smelting or electroplating, and the use of fertilizer in agricultural fields. Excess Cd may cause renal failure and the softening of bones as a result of long-term or high-dose-contamination exposure, and prostate cancer may develop in response to high levels of Cd [29,30].

In our study, the concentration of Zn was higher than those of other metals in the examined fish and shellfish muscle (Table 2). However, the Zn concentration in this study was lower than the findings of Hossain et al. [20]. Further, this did not exceed the permissible limit in fish and shellfish muscle. Zn is an essential micronutrient for living organisms and acts as a catalyst for about 300 enzymes in aquatic creatures. Therefore, a relatively high level of Zn is required to balance certain biological functions. Zn is involved in most metabolic pathways in humans, and its insufficiency can lead to loss of appetite, the inhibition of growth, skin changes, and immunological abnormalities [31].

In this research, the Cu level in fish and muscle exceeded the guideline value for Bangladesh. In addition, the values of Cu were lower than the findings of Hossain et al. [20]. Due to its presence in several enzymes and its necessity for hemoglobin synthesis, it is known as an essential element [32]. High levels of copper (Cu) can cause acute toxicity. In this study, the highest level of Cu was found in the body of *Penaeus monodon* (42.33 µg/g), and the lowest concentration was found in the body of *Labeo bata* (2.93 µg/g). However, Cu in the aquatic environment can originate from dyeing and tanning industries, textiles, photography, paints and inks, the battery industry, and surface runoff from upstream agricultural fields [14,33,34]. The concentration of Ni in the environment is very low, and it causes a variety of adverse health effects, such as lung inflammation, fibrosis, emphysema, and tumors. The highest concentration of Ni was found in the body of *Labeo bata* (0.81 mg/kg), and the lowest concentration was reported in the body of *Sillaginopsis penigus* (0.02 µg/g).

### 3.2. Health Risk to Fish and Shrimp Consumers

#### 3.2.1. Estimated Daily Intake (EDI)

It is necessary to determine the toxic potency of these metals, which depends on exposure doses. In this regard, comparing the concentrations of the metals with the estimated daily intake limits was used as an active tool to evaluate the balance between benefits and risks in this study. The estimation of the daily intake of the metals is based on the concentrations of the metals in fish and shrimp muscles. The recommended daily allowance (RDA) of the elements Pb, Cd, Cr, Ni, Cu, and Zn was set by the WHO [35] as 0.25, 0.07, 0.23, 2–5, 0.9, and 11 µg/g/person, respectively. In this study, the average EDIs of Pb, Cd, Ni, Cu, and Zn were 5.61 × 10^−4^, 4.96 × 10^−6^, 4.33 × 10^−3^, 2.2 × 10^−3^, and 3.68 × 10^−3^ (Table 3).

The mean EDI values of the metals were lower than RDA (Recommended Daily Allowance) standards, indicating the low risk of these metals to consumer health. However, it would be unwise to utilize this method as the sole measure to establish a clear determination.

#### 3.2.2. Target Hazard Quotient (THQ) and Hazard Index (HI) Value of Fish and Shrimp

If THQ is less than 1, the affected person is less likely to experience detrimental impacts; if it is greater than 1, there may be a possibility of potential health risks (Wang et al., 2005). The average individual THQ values of Pb, Cd, Ni, Cu, and Zn in this study were 0.641, 0.0216, 0.0125, 0.814, and 0.173. The highest value of THQ is shown in the metal Ni. All of the values of THQ are below 1, which suggests no adverse health effects (Table 4). The HI or Total THQ (TTHQ) from the fish was 0.42, which is also below 1 and also proves that there are no adverse health effects [1,12,22]. The highest HI value (0.78) was found for *L. bata*, and the lowest (0.24) was obtained for *P. fuscus.*

However, the long-term and/or excessive consumption of fish and shrimp species may have non-carcinogenic health effects. Although the analysis of THQ for human health risk assessments has no dose–response relation to the studied elements, multiple simultaneous pollutants can cause severe harm to humans.

### 3.3. Source Identification

Strong correlations between specific heavy metals may indicate similar types of pollution and/or release from the same source of contamination, mutual influence, and equivalent behavior when being transported to the aquatic system [1,12,16,22]. A correlation matrix among the metals from fish species is presented in Table 5. The correlation matrix analyzes whether there are any interrelationships among the elements. The correlation can be positive or negative and significant or highly significant (Table 5). The presence of a correlation between metals indicates that they have originated from similar sources, and no correlation implies different sources or diverse sources. A negative correlation was observed between Ni and Pb (*r* = −0.816); positive correlations were found between Cu and Pb (*r* = 0.623), Zn and Cu (*r* = −0.871), and Zn and Cu (*r* = 0.498); and very weak or no correlations were calculated between Pb and Cd, Ni and Zn, and Pb and Zn. The correlations among Ni, Pb, Zn, and Cu indicate they originated from anthropogenic sources. The study area and its catchment are mainly used for agriculture activities, poultry farming, fish farming, small industries such as food processing and packaging, still workshops, ship and boat building, oil refineries, cement industries, electroplating, etc. (Islam et al., 2018 [14]; Tahity et al., 2022 [12]). Cd and Ni are generally used in nickel–cadmium batteries, foils, alloys, and electroplating (Islam et. al., 2018 [14]). The battery industry employs Cd and generates and discharges wastes with high Cd levels. Pb, on the other hand, has a long history of being employed as an anti-corrosive element in paint for steel mills and boats. Pb is also released via engine oil leakages from boats and ships. The burning of fuels also possibly contributes to the increased level of Pb. Although naturally available, advective transport through the river and atmospheric deposition might have contributed to the levels of Cu and Zn.

**Figure 3:**
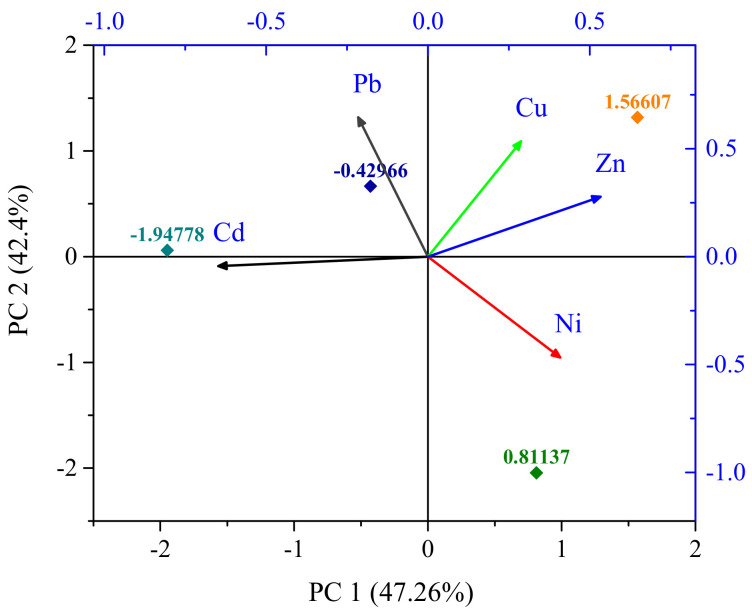
Loadings of PCA of the studied metals in samples.

PCA identifies a compressed set of factors that affect the variance in an entire data set [36]. Hence, PCA was performed for the qualitative analysis of the clustering behavior [37]. The results included the corresponding loading values, along with the particular eigenvalues, the variance in 100% of the data, and the cumulative variance for each factor, and two elements evolved with the eigenvalues, which were greater than 1 (Table 6). Figure 3 and Table 2 show the PCA outcomes with two grouping factors and a variance of 89.66%; the results show that PC1 provided the highest eigenvalue, 2.36, which was dominant, and a highly significant factor group contributing 47.26% of the total variance with the loading of Zn (*r* = 0.533). The findings specified that industrial and agricultural chemicals were probable sources [38,39]. Meanwhile, PC2 contributed to 42.40% of the total variance in the loads of Pb (*r* = 0.645) and Cu (0.535). The source of Pb and Cu might be urban or industrial wastes and other non-geogenic activities and electroplating actions, respectively [22]. 

**Figure 4:**
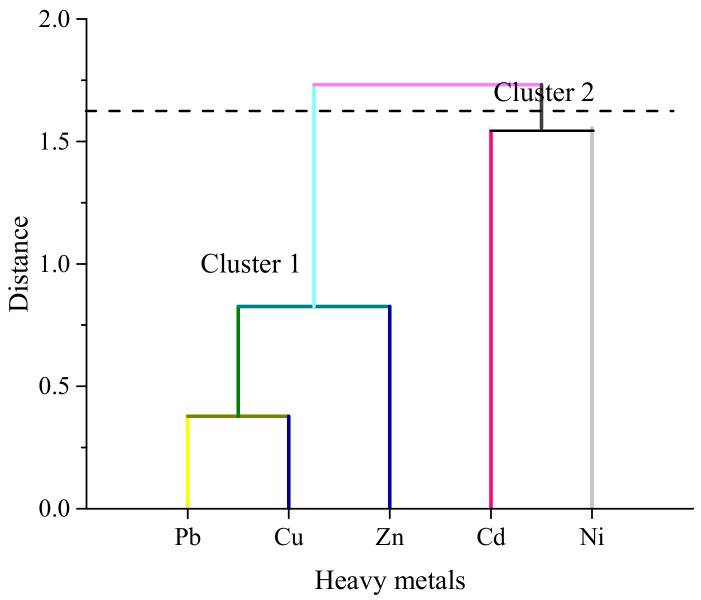
Hierarchical clusters (dendrogram) of the metals in the samples.

Cluster analysis is mostly used for the presentation of similar groups of the sampling site that have special variability. Similar groups of sites are presented as a group of clusters, and dissimilar sites are plotted in another group of clusters to identify specific areas in terms of contamination [39,40,41]. A hierarchical agglomerative cluster analysis was performed on the normalized data set using Ward’s method with Euclidean distance as a measurement of similarity (Figure 4). The cluster analysis resulted in a dendrogram with a significant cluster at (Dlink/Dmax) × 100 = 20. Two major groups were identified: Pb, Cu, and Zn in cluster 1 and Cd and Ni in cluster 2, demonstrating their close association and possibly indicating the same sources of origin.

## 4. Conclusions

This study evaluated the accumulation and risk levels of six toxic metals (Pb, Cd, Cr, Ni, Cu, and Zn) in four fish and shrimp samples from the central coast of Bangladesh. According to the findings, only Pb in fin fish and Pb, Cu, and Zn in shrimp samples exceeded the guideline values for Bangladesh, representing possible risks to consumers. Surprisingly, Pb concentrations were 6.5 times higher in shrimp samples. The risk indices (EDI, THQ, and HI) showed no carcinogenic health risks to Bangladeshi coastal people; however, without considering the bioavailability of the metals in the human body, it cannot be concluded that there is no risk for future consumption. The close associations, as revealed by the correlation matrix, between Cu and Pb and between Zn and Cu indicate the same sources of origin. Multivariate analyses, cluster analysis (CA), and principal component analysis (PCA) also demonstrated the possibility of the same anthropogenic sources of Pb, Cu, and Zn in the study area, specifically industrial wastes and agricultural chemicals. Therefore, it is advised to take appropriate control measures and to constantly monitor the presence of these harmful metals in fish species.

## Figures and Tables

**Table 1:** Analysis of Certified Reference Material for fish.

Metals	Certified Metal Value, (mg/kg)	Average Recovery from Diluted Solution (%)
Pb	10.0 ± 0.5	103
Cd	5.0 ± 0.5	102
Cr	50 ± 0.16	102
Ni	0.93 ± 0.12	97
Cu	50.0 ± 0.5	100
Zn	100.0 ± 0.5	97

**Table 2:** Comparison of heavy metals (mean ± SD) in fish and shrimp muscles with various standard levels and relevant studies (µg/g) (*n* = 48).

Fish								
Sampling Site	Species	Pb	Cd	Cr	Ni	Cu	Zn	Reference
Central coast	*L. bata*	3.53 ± 0.56	0.05 ± 0.01	BDL	0.81 ± 0.02	2.93 ± 0.2	39.61 ± 2.97	Present study
	*P. fuscus*	7.17 ± 1.08	0.08 ± 0.03	BDL	0.19 ± 0.017	14.88 ± 2.19	27.75 ± 1.63	
	*S. panijus*	6.95 ± 0.97	0.06 ± 0.012	BDL	0.02 ± 0.01	11.16 ± 1.23	45.89 ± 2.78	
Central coast	*L. calcarifer*	1.33 ± 0.30	0.05 ± 0.03	0.42 ± 0.32	NA	0.50 ± 0.12	2.40 ± 0.40	[12]
Noakhali	*Otolothoides pama*	0.38 ± 0.12	NA	9.69 ± 2.04	BDL	30.29 ± 3.93	107.22 ± 16.87	[20]
	*Apocryptes bato*	0.65 ± 0.03	NA	7.35 ± 3.18	BDL	46.98 ± 24.22	111.81 ± 6.37	
	*P. paradiseus*	0.48 ± 0.07	NA	7.24 ± 3.59	BDL	33.95 ± 4.14	101.38 ± 8.38	
	*Mystus gulio*	0.57 ± 0.04	NA	BDL	BDL	46.57 ± 12.39	129.33 ± 13.45	
	*Harpadon nehereus*	0.20 ± 0.02	NA	BDL	BDL	35.40 ± 17.83	106.72 ± 6.02	
	*Lates calcarifer*	0.44 ± 0.05	NA	8.84 ± 0.12	BDL	31.44 ± 2.97	103.72 ± 13.81	
Patuakhali	*Tenualosa ilisha*	0.5 ± 0.5	0.2 ± 0.2	0.5 ± 0.2	0.5 ± 0.4	1.1 ± 0.6	NA	[21]
Chittagong	*O. pama*	0.14 ± 0.028	NA	1.525 ± 0.049	0.225 ± 0.007	NA	31.543 ± 1.275	[22]
	*Polynemus paradiseus*	0.165 ± 0.02	NA	1.036 ± 0.005	0.345 ± 0.007	NA	33.24 ± 0.98	
	*Apocryptes bato*	0.086 ± 0.008	NA	0.709 ± 0.014	0.255 ± 0.007	NA	1458.7 ± 1.42	
Tolerance level in fish		0.5	0.5	0.15	-	30	40	[23]
Guidelines for Bangladesh		0.3	0.25	1	-	5	50	[24]
**Shrimp**								
Central coast	*P. monodon*	7.24 ± 0.95	0.04	BDL	0.46 ± 0.03	42.33 ± 2.03	49.98 ± 2.09	Present study
Chittagong	*Macrobrachium rosenbergii*	0.025 ± 0.002	NA	1.17 ± 0.212	0.325 ± 0.007	NA	70.34 ± 1.26	[22]
	*Metapenaeus dobsoni*	0.056 ± 0.008	NA	1.25 ± 0.036	0.34 ± 0.000	NA	69.05 ± 0.41	
Cox’s Bazar	*P. monodon*	17.75 ± 1.5	0.09 ± 0.03	0.69 ± 0.6	NA	9.43 ± 2.8	18.89 ± 2.9	[1]
Saint Martin	*P. sculptilis*	0.69 ± 1.56	0.713 ± 0.06	< 0.08	NA	5.049 ± 0.07	13.5 ± 0.43	[5]
	*P. versicolor*	< 0.3	3.505 ± 0.19	< 0.08	NA	13.398 ± 0.45	22.413 ± 0.35	
Tolerance level in crustacean		0.5	0.5	0.5	-	5	50	[25]
Guideline for Bangladesh		0.5	0.5	1	-	5	50	[24]

BDL = below detection level; NA = not assessed.

**Table 3:** EDI concentrations (µg/g/person) of fish and shrimp.

Name of the Species	Pb	Cd	Cr	Ni	Cu	Zn
*L. bata*	3.18 × 10^−4^	4.51 × 10^−6^	BDL	7.30 × 10^−5^	2.64 × 10^−4^	3.57 × 10^−4^
*P. fuscus*	6.46 × 10^−4^	7.21 × 10^−6^	BDL	1.72 × 10^−2^	1.34 × 10^−3^	2.51 × 10^−3^
*S. penijus*	6.26 × 10^−4^	5.41 × 10^−6^	BDL	1.81 × 10^−6^	1.00 × 10^−3^	4.14 × 10^−3^
*P. monodon*	6.53 × 10^−4^	4.51 × 10^−6^	BDL	4.15 × 10^−5^	3.82 × 10^−3^	4.51 × 10^−3^

**Table 4:** THQ values of fish and shrimp.

Name of the Species	Pb	Cd	Ni	Cu	Zn	TTHQ/HI
*L. bata*	9.08 × 10^−2^	4.51 × 10^−3^	6.64 × 10^−3^	6.60 × 10^−1^	1.19 × 10^−2^	0.774
*P. fuscus*	1.85 × 10^−1^	7.21 × 10^−3^	1.92 × 10^−3^	3.35 × 10^−2^	8.37 × 10^−3^	0.236
*S. penijus*	1.79 × 10^−1^	5.41 × 10^−3^	1.51 × 10^−4^	2.5 × 10^−2^	1.38 × 10^−1^	0.384
*P. monodon*	1.86 × 10^−1^	4.51 × 10^−3^	3.77 × 10^−3^	9.55 × 10^−2^	1.51 × 10^−2^	0.305
Average	6.41 × 10^−1^	2.16 × 10^−2^	1.25 × 10^−2^	8.14 × 10^−1^	1.73 × 10^−1^	0.416

**Table 5:** Pearson correlation matrix among heavy metals (*n* = 48).

	Pb	Cd	Ni	Cu	Zn
Pb	1				
Cd	0.277	1			
Ni	**−0.816**	−0.555	1		
Cu	**0.623**	−0.457	−0.084	1	
Zn	0.074	**−0.871**	0.087	0.498	1

Significant values are marked in bold font (*p* ≤ 0.05).

**Table 6:** Principal component analysis for the metals.

	PC 1	PC 2
Pb	−0.216	0.645
Cd	−0.647	−0.043
Ni	0.409	−0.467
Cu	0.288	0.535
Zn	0.533	0.278

## Data Availability

The data are provided in the article.

## Supplementary Materials

The following supporting information can be downloaded at: https://www.mdpi.com/article/10.3390/biology11121780/s1, Table S1: Water quality parameters of the study area after Begum et al. [42].
