# Supplementary material for: Heavy Metals in Four Marine Fish and Shrimp Species from a Subtropical Coastal Area: Accumulation and Consumer Health Risk Assessment
Source: Biology (Basel). 2022 Dec 7;11(12):1780. doi: 10.3390/biology11121780 (PMC9775417; doi:10.3390/biology11121780)
Supplement: Supplementary file 1 [file biology-11-01780-s001.zip › biology-1907310-supplementary.pdf]

Table S1: Water quality parameters of the study area after Begum et al. [1]

1	Parameters	Range
2	pH	7.72 to 7.91
3	Temperature	19.1 to 19.6 °C
4	Salinity	0.5 to 17 ppt
5	Hardness	94 to 126 mg/L
6	DO	3.33 to 4.3 mg/L
7	BOD	1.23 to 2.9 mg/L
8	Conductivity	525 to 714 µS/cm
9	TDS	113 to 197.67 mg/L

1. Begum, S.; Islam, M.S.; Rehnuma, M.; Kabir, M.H.; Tusher, T.R. Water quality and fish diversity status in Meghna River at Narsingdi in Bangladesh. *Bangladesh J. Environ. Sci*. **2019**, *37*, 25–31.
